# Washington City Dental Society

**Published:** 1882-01

**Authors:** 


*Washington City Dental Society.*—The annual meeting of this Society was held in Washington, December 13th, 1881. The following officers were elected to serve for the ensuing year:

President: R. Finley Hunt.
Vice President: J. Curtiss Smithe.
Recording Secretary: H. M. Schooley.
Reporting Secretary: H. B. Noble.
Treasurer: R. B. Donaldson.
Librarian: L. C F. Hugo.
To deliver the annual address: J. B. Hodgkin.

This Washington City Society is an active, energetic and harmonious body, and this last is saying a good deal in this day of professional bickering and cynical criticism. We have never heard of a split or dissention among its members. H.

---

